# Lessons Learned from Reconstructing Severe Hand Injuries During the COVID-19 Pandemic

**DOI:** 10.3390/jcm14072169

**Published:** 2025-03-22

**Authors:** Christina Glisic, Tonatiuh Flores, Erol Konul, Hugo Sabitzer, Giovanni Bartellas, Alexander Rohrbacher, Berfin Sakar, Sascha Klee, Uwe Graichen, Patrick Platzer, Klaus F. Schrögendorfer, Konstantin Bergmeister

**Affiliations:** 1 Karl Landsteiner University of Health Sciences, Dr. Karl-Dorrek-Straße 30, 3500 Krems, Austriauwe.graichen@kl.ac.at (U.G.); konstantin.bergmeister@stpoelten.lknoe.at (K.B.); 2 Clinical Department of Plastic, Aesthetic and Reconstructive Surgery, University Clinic of St. Poelten, Dunant-Platz 1, 3100 St. Poelten, Austria; 3 Clinical Department of Orthopedics and Traumatology, University Clinic of St. Poelten, Dunantplatz 1, 3100 St. Poelten, Austria; 4 Clinical Laboratory for Bionic Extremity Reconstruction, University Clinic for Plastic, Reconstructive and Aesthetic Surgery, Medical University of Vienna, 1090 Vienna, Austria

**Keywords:** hand injuries, hand surgery, COVID-19 pandemic, trauma, micro amputation injuries

## Abstract

**Background**: COVID-19 presented many challenges for our health system, one being a suspected change in the epidemiology of severe hand trauma modalities. These complex injuries are traditionally treated at specialized hand trauma centers, but COVID-19 has in many ways disturbed these established pathways and presented new challenges. **Methods**: We retrospectively analyzed finger amputation injuries treated at the University Hospital of St. Poelten between 2018 and 2022 to examine differences in the management of micro amputation injuries before and during the COVID-19 pandemic. Further challenges in the treatment of hand trauma patients were analyzed and solutions were developed. **Results**: Overall, the number of occupational finger amputation injuries in Lower Austria declined during the COVID-19 pandemic. Contrarily, more private accidents were treated in the same period, suggesting a lockdown specific change in injury characteristics. Throughout the entire examined period, a total of 130 injured fingers, including 29 thumbs, were treated. In 67 cases, a reconstruction attempt was feasible and successful in 59 cases. Specific challenges were fewer active hand trauma centers, subsequent long transport times, specific COVID-19 prevention measures, and limited postoperative rehabilitation resources. **Conclusions**: Despite many challenges overall affecting the time to revascularization, good results were achieved by small but meaningful modifications. These included well-established principles such as back table preparation and strengthening novel concepts such as tele-medicine for patient selection. Overall, the reconstruction of severe hand injuries is often challenging, especially during a world-wide health crisis, but with adequate solutions, good results can be readily achieved.

## 1. Introduction

Severe hand injuries remain a surgical challenge for physicians and healthcare providers. Their aftermath often entails severe social and economic consequences [1]. Such injuries have traditionally been treated at specialized hand trauma centers to provide high-quality short- and long-term treatment. Thereby, advancements have been made in reconstructing complex hand injuries in recent decades, since the first successful digit replantation in 1968 by Komatsu and Tamai [2]. While the demand on resources to provide such 24/7 services is high, studies have shown the cost-effectiveness and the better functional outcome of microsurgical hand reconstruction compared to low-tech solutions [3,4].

The COVID-19 pandemic has presented many novel challenges and alterations for our health systems and thereby also changed the treatment of hand cases. Operation capacities were shut down completely, except for acute burn injuries, tumor surgery, acute trauma, and other urgent surgeries in hospitals around the globe [5]. Additionally, the number of elective hand surgeries was decreased tremendously, while emergency surgeries increased [6]. In particular, domestic injuries rose in numbers [7,8,9,10].

To adapt to this challenging time, active surgical centers established, for example, positive pressure operation rooms and telemedicine during the pandemic to reduce COVID-19 transmission and provide continuous service [11,12,13].

Many coping modalities during the COVID-19 pandemic have been published, yet only a few focus on managing acute minor amputations. Thus, we provide a thorough investigation of the challenges of treating complex hand injuries at a maximum care hospital. Given the risk of other global menaces and the possibility of another health system collapse, we share our personal experiences in acute trauma reconstruction during this period of a world-wide health crisis.

## 2. Materials and Methods

### 2.1. Study Design

We retrospectively analyzed micro amputation injuries treated at the clinical department of plastic, aesthetic, and reconstructive surgery at the University Hospital of St. Poelten during the period from 1 January 2018 to 31 May 2022. This period was divided into two periods of equal length. The pre-COVID-19 period covered from 1 January 2018 to 15 March 2020 and the COVID-19 period covered from 16 March 2020 to 31 May 2022. These time periods were selected as the initial nationwide lockdown in Austria commenced on 16 March 2020, imposing substantial restrictions on daily activities. The two groups were compared to examine possible differences in cause of injury, treatment, and frequency of finger amputation injuries. We defined a total amputation as an amputation without hard or soft tissue connection, and a subtotal amputation with the main blood vessels severed and a hard or soft tissue connection with less than one-quarter of the circumference. In our study, a successful replantation was defined as survival of the replant beyond at least 14 days.

In our study, we included patients with finger amputation injuries (male and female, at any age) eligible for reconstruction during the period from 1 January 2018 to 31 May 2022 who received treatment and follow-up treatment at the University Hospital of St. Poelten.

Patients with severe trauma that prohibited a reconstructive attempt and patients who died prior to their arrival at the hospital were excluded.

Ethical approval was acquired from the local institutional review board at Karl Landsteiner University of Health Sciences Krems, Austria (reference number: 1001/2022).

The following parameters were collected: patient characteristics (age, gender), trauma characteristics (injured anatomical structures, characteristics of the accident), operative procedures, in-hospital treatment, postoperative complications (venous insufficiency, secondary amputation), and follow-up.

Furthermore, challenges such as the risk of a COVID-19 infection or long ischemia time due to COVID-19 prevention and others faced during the COVID-19 pandemic were analyzed and specific solution strategies were elaborated on to enable an improved treatment of micro amputation injuries in the future.

### 2.2. Statistics and Data Management

All data were documented pseudonymously, and data protection management complied with Austrian legislation. Data collection was performed using Microsoft Excel (© 2023 Microsoft, Redmond, WA, USA, version 16.77.1), and statistical analyses were performed using IBM SPSS Statistics (© IBM, Armonk, NY, USA, version 29) and Gnu R (version 4.3.2). Nominal data were described using percentages and absolute frequencies. For continuous variables, such as age, the normality of the distribution was first assessed using the Shapiro–Wilk test. Based on the results of the normality test, appropriate statistical methods were applied. For normally distributed metric data, the mean and the standard deviation were used. However, since age was not normally distributed in the cohorts under consideration, as determined by the Shapiro–Wilk test, the Wilcoxon rank-sum test was employed to analyze the age differences between the pre-COVID-19 and COVID-19 periods. For categorial data, Fisher’s exact test was used when there were five or fewer occurrences per category, and the chi-squared test for count data was applied in all other cases. The empirical effect size was quantified using Cramér’s V.

## 3. Results

**Figure 1:**
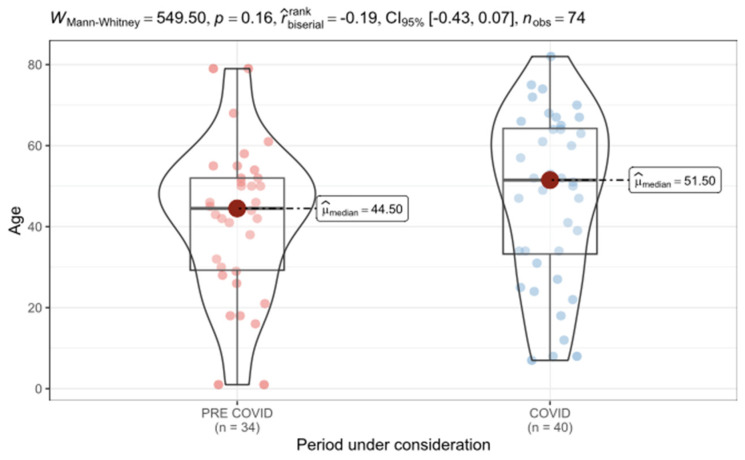
Age distribution of patients with finger amputations injuries during the pre-COVID-19 (red) and the COVID-19 (blue) period.

**Figure 2:**
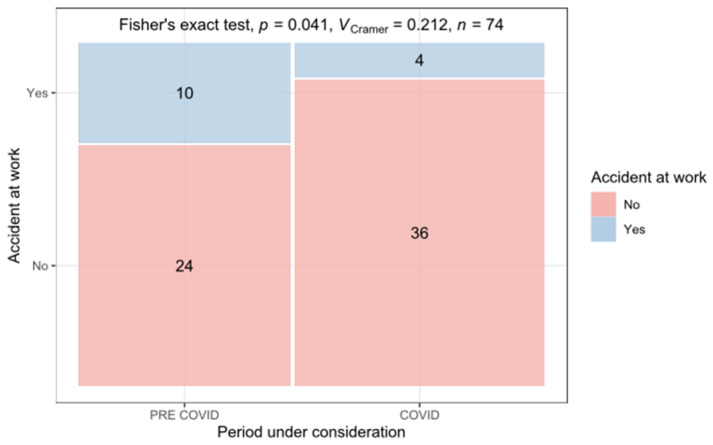
Number of work-related injuries (blue) during the pre-COVID-19 (**left**) and the COVID-19 period (**right**).

In total, 130 micro amputation injuries in 74 patients were treated between 1 January 2018 and 31 May 2022. Here, 67 (91%) patients were male and 7 (9%) were female. The patients’ mean age was 48 years, ranging from 1 to 82 years at the time of injury (Figure 1). The mean age of the treated patients did not rise significantly from 44.5 years pre-COVID-19 to 51.5 years during the pandemic. However, the number of work-related injuries declined significantly from 29% during the pre-COVID-19 period to 10% during the COVID-19 period (Figure 2).

### 3.1. Trauma Characteristics

In 35 (47%) patients, the right hand was injured, while the left hand was injured in 39 (53%) patients. There was a significant difference before and during the pandemic. During the pre-COVID-19 period, the right hand was affected in 62% of cases. Unexpectedly, this number decreased to only 35% during the COVID-19 period.

**Figure 3:**
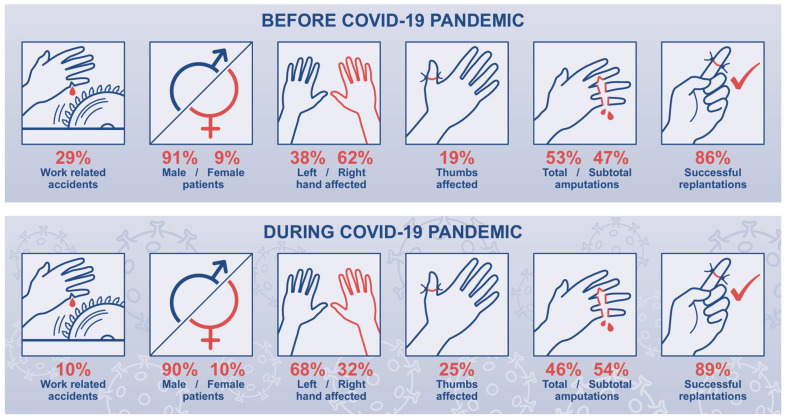
Data concerning finger amputation injuries before the COVID-19 pandemic (**above**) and during the COVID-19 pandemic (**below**).

In total, 130 fingers (including thumbs) were injured. Out of these, 64 were total and 66 were subtotal amputations. Here, 29 thumbs were amputated. Thus, there were eleven total and eighteen subtotal amputations (Figure 3).

### 3.2. Anatomical Zones of Injury

Comparing amputation levels before and during the COVID-19 pandemic shows similar trends. Pre-COVID-19, 22 cases (16.9%) occurred at the MCP/proximal phalanx, while during the COVID-19 pandemic, we saw 21 cases (16.2%). At the PIP/intermediate phalanx, there were 22 cases (16.9%) pre-COVID-19 and 23 cases (17.7%) during the COVID-19 pandemic. Amputations at the distal interphalangeal joint/distal phalanx accounted for 11 cases (8.5%) pre-COVID-19 and 15 cases (11.5%) during the COVID-19 pandemic. Injuries at the distal interphalangeal joint accounted for nine cases (6.9%) pre-COVID-19 and seven cases (5.4%) during the COVID-19 pandemic (Table 1).

### 3.3. Reconstruction/Surgery

In 63 (48%) fingers in total, microsurgical exploration in the OR demonstrated such severe vessel or bone trauma that a reconstruction was not feasible, so digit amputation was performed. There was no statistical difference in the number of non-feasible finger replantations comparing the pre-COVID-19 and the COVID-19 groups. In the remaining 67 (52%) cases, a reconstruction attempt was indicated and conducted. Here, in 59 cases, reconstruction was successful, demonstrating a success rate of 88%. For the remaining eight fingers, secondary amputations had to be performed. The reasons for secondary amputation were venous insufficiency and an improper handling of amputation fingers before replantation.

### 3.4. Treatment Characteristics and Postoperative Care

After surgery, the patients received occupational therapy by default adjusted to the degree of injury and wound healing status. During the hospital stay, occupational therapy at our hospital was permanently accessible without restrictions. After discharge, specialized outpatient hand therapy was required in all replanted cases to improve and sustain functional convalescence. Yet, the availability of physical hand therapy and occupational therapy was problematic as many therapy practices were not open due to the pandemic. This problem was more pronounced in rural regions, with less accessibility of hand therapists.

### 3.5. COVID-19-Specific Challenges and Solutions

Due to the COVID-19 pandemic, many challenges were encountered. The risk of COVID-19 infection was one of the most infringing threats throughout the period until the patient arrived at the operation room. This included transmission both among patients and between medical staff and patients. Direct transfer from the emergency room to the operating room and preventive measures such as wearing masks and additional protective clothing were instrumental in mitigating this risk.

Further, another challenge was long ischemia time, which emerged when attempting to prevent COVID-19 (i.e., waiting for the COVID-19 test result). Every minute was used wisely using back table preparation in a second operation room in amputation injuries.

Additionally, the high reconstructive demand due to fewer active trauma centers was a significant challenge. A pre-selection process was conducted where the patient’s health condition, comorbidities, the extent of the injury, and the patient’s preference concerning a reconstruction attempt were discussed via telemedicine. Further, X-rays and photographs were sent to hand trauma centers in advance. Thus, time to treatment was minimized.

**Figure 4:**
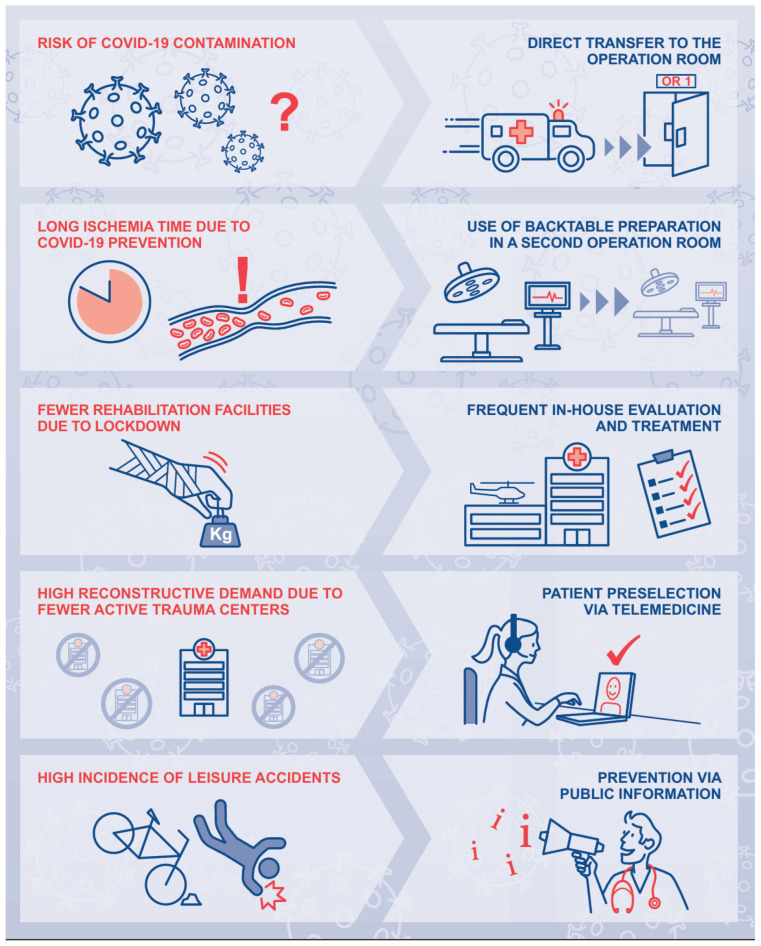
COVID-19-specific challenges (**left**) and developed solutions (**right**).

And lastly, the high incidents of leisure accidents, as mentioned above, were noticeable higher during the lockdown periods. Therefore, we highly recommend extending public information to prevent severe hand injuries in the future (Figure 4).

### 3.6. Limitations

A limitation of this study was the inability to precisely determine ischemia time in the majority of cases due to inadequate preclinical documentation as patients had to cover varying distances to reach their primary care facility and were transferred to our facility after a primary examination. Additionally, due to the patients’ shock state in some cases, it was not possible to determine the exact time of the accident. However, it can be stated that in all cases, the duration was estimated to be under 8 h.

## 4. Discussion

Fortunately, the COVID-19 pandemic, along with its many challenges for reconstructive hand surgery, has passed. In retrospect, viable lessons can be learned from this time of global crisis to better address similar situations in the future. 

In our study, we show that a high rate of limb survival above 85% was achievable despite complicating factors such as prolonged transport, time to intubate, and consequently ischemia time. Many of these challenges arose from the need to minimize COVID-19 infections among healthcare workers and thus prevent high-risk situations, for example, during intubation for the OR personnel preparing for surgery. Therefore, established routine workflows to readily get patients in the OR and provide a timely blood flow to severed extremities were disturbed. In addition, the transport from the site of the accident to a replantation center such as our department was often delayed due to limited resources and again the need to prevent infection among paramedics and hospital personnel. Although detailed information regarding transmission between trauma patients and our personnel was not available, we had a surprisingly low COVID-19 infection rate throughout the pandemic and its lockdowns, reflecting the success of our preventive measures. Consequently, our replantation service was available throughout the whole pandemic. 

To conquer these new challenges, we adapted to the established principles of replantation surgery and developed new concepts. For example, during the arrival of the patient in the operation room and intubation, no other personnel were allowed in the OR; therefore, preparation of the OR instruments and the amputated extremities was performed in an adjacent OR for “back table preparation”. Furthermore, patients referred to us secondarily from another hospital were first screened thoroughly if replantation was feasible before their transport to our center was initiated. Then, the patient and the trauma were analyzed via telemedicine to determine the health state of the patient, the injury mechanism, and the clinical situation. In many cases, X-rays and clinical photographs were then accessed via the telemedical system to complete the evaluation. Therefore, patients who were either not suitable for prolonged surgery or where reconstruction was prohibited due to the sustained injury were treated at the primary hospital, if possible, via amputation. In that way, our service remained open for new arrivals while the quality of care for patients was maintained, and injuries were treated as quickly as possible. Interestingly, the epidemiology of severe hand injuries changed significantly during the COVID-19 pandemic. Overall, the number of work-related injuries dropped, and leisure accidents were more prevalent than before. Although not specifically evaluated, many patients involved in leisure accidents described using their lockdowns to carry out DIY projects such as home improvements. Some authors from different countries recognized an increased number of DIY/domestic injuries, while the number of work-related injuries decreased according to other authors [7,8,9,10,14,15]. This could be explained by regulations (i.e., curfew or home office) due to the pandemic. Also, spending more time at home resulted in, for example, making home improvements, where patients were using tools they had potentially never used before. Using unfamiliar tools and conducting novel home improvements have previously been described as significant risk factors for hand injuries [16].

However, the typical patient with severe hand trauma remained similar: a middle- aged, right-handed man injuring their non-dominant left hand. This finding from a Lower Austrian population has also been seen in other countries, for example, in Australia [7]. Perhaps previously successful efforts in informing the public on how to prevent these severe injuries should also be considered in the event of future pandemics.

Finally, successful surgery is only one part of the reconstructive effort in hand injuries. Additionally, thorough postoperative rehabilitation is crucial to obtain a positive result. During the pandemic, the immediate postoperative treatments for inpatients, for example, changing dressings, wound inspections, occupational treatments, or physiotherapy, took place as before the pandemic without any restrictions. However, outpatient treatment was often severely limited, especially in more rural locations. As we receive patients from a broad catchment area, some patients live far away from our clinic or other institutions that offer these postoperative treatments. The lockdown impaired treatment, as many smaller or private facilities were closed, or had reduced capabilities throughout the pandemic. Therefore, less treatment slots were available, and the postoperative rehabilitation was sometimes not ideal. We tried to solve this problem with prolonged in-house treatment where necessary and frequent outpatient evaluations to identify prolonged healing or rehabilitation early on. Hereby, we were able to provide a high-quality service during this challenging period and obtained good results. While we hope another pandemic will not come any time soon, we believe that much has been learned during this difficult time.

## 5. Conclusions

In conclusion, severe hand injuries remain a reconstructive challenge. Here, we show COVID-19 pandemic-specific problems and how we were able to solve these while maintaining a high-quality standard. Overall, small but crucial improvements have helped to rehabilitate affected patients back to their independence.

## Figures and Tables

**Table 1:** Anatomical characteristics of hand injuries pre-COVID 19 and during COVID-19.

	Pre-COVID-19	COVID-19
**MCP/prox. phalanx**	22 (16.9%)	21 (16.2%)
**PIP/intermediate phalanx**	22 (16.9%)	23 (17.7%)
**DIP/distal phalanx**	11 (8.5%)	15 (11.5%)
**IP/distal phalanx**	9 (6.9%)	7 (5.4%)

## Data Availability

All the data analyzed during the current study are available from the corresponding author upon reasonable request.
